# Spatiotemporal Regulation of Hsp90–Ligand Complex Leads to Immune Activation

**DOI:** 10.3389/fimmu.2016.00201

**Published:** 2016-05-24

**Authors:** Yasuaki Tamura, Akihiro Yoneda, Norio Takei, Kaori Sawada

**Affiliations:** ^1^Department of Molecular Therapeutics, Center for Food and Medical Innovation, Institute for Innovation and Business Promotion, Hokkaido University, Sapporo, Japan

**Keywords:** heat shock protein, danger signal, cross-presentation, dendritic cell, toll-like receptor, autoimmune disease

## Abstract

Although heat shock proteins (HSPs) primarily play a pivotal role in the maintenance of cellular homeostasis while reducing extracellular as well as intracellular stresses, their role in immunologically relevant scenarios, including activation of innate immunity as danger signals, antitumor immunity, and autoimmune diseases, is now gaining much attention. The most prominent feature of HSPs is that they function both in their own and as an HSP–ligand complex. We here show as a unique feature of extracellular HSPs that they target chaperoned molecules into a particular endosomal compartment of dendritic cells, thereby inducing innate and adaptive immune responses via spatiotemporal regulation.

## Introduction

Heat shock proteins (HSPs) are known to act as molecular chaperones within cells. They are primarily considered to be intracellular proteins that have protective functions under cellular stress conditions. Recently, the existence of extracellular HSPs has been shown, and much attention has been paid to their role in stimulation of innate and adaptive immunity. Extracellular HSPs have been shown to activate innate immune responses through toll-like receptors (TLRs) and scavenger receptors (SRs) expressed on antigen-presenting cells (APCs), such as dendritic cells (DCs) and macrophages (1). Moreover, it has been demonstrated that extracellular HSPs augmented the ability of their associated molecules to activate immune responses by efficient targeting to antigen-presenting cells (2).

It is well known that immunization with an HSP–peptide complex is able to elicit peptide-specific T cell responses (2–6). However, the behavior of an HSP–peptide complex after uptake by APCs has not been completely elucidated. Presentation of an exogenous antigen to CD8^+^ T cells is called cross-presentation (7). Cross-presentation is a process by which APCs, including DCs, uptake pathogens and dying cellular fragments and present proteolytic fragments derived from these antigens in the context of MHC class I molecules (8). This process is a fundamental mechanism of the induction of antitumor immune responses. However, it is not known how antigens are taken up and where are they destined to go and encounter MHC class I molecules. We have uncovered intracellular pathways that link the antigen internalization pathways and their processing as well as loading on MHC class I molecules (9–12). Antigenic peptides chaperoned by extracellular Hsp90 or the Hsp70 family member ORP150 are targeted to static early endosomes and processed by endosomal peptidases, followed by loading onto MHC class I. By contrast, HSP-chaperoned proteins that are required for proteasomal degradation enter both the endosomal pathway and proteasome–TAP-dependent pathway (11). Moreover, it is thought that HSP receptor-expressing APCs play a key role in the targeting of an HSP–antigen complex into these cross-presentation pathways (13, 14). To begin with, we will describe the history and development of HSP in tumor immunology. Then, we will discuss the emerging roles of extracellular HSPs in the regulation of innate immunity and adaptive immunity with focus on how spatiotemporal regulation of HSP–ligand complexes within antigen-presenting cells affects immune responses.

## HSPs in Cancer Immunobiology: Orchestration of Innate Immunity and Adaptive Immunity

### HSPs as Tumor Antigens

Heat shock proteins are generally considered to be intracellular chaperones that are essential for maintaining cellular homeostasis. From an immunological point of view, much attention has been paid to emerging roles of extracellular HSPs as endogenous immunomodulators for innate and adaptive immune responses. Pioneering studies by Srivastava and colleagues first demonstrated tumor-specific antigenicity to Gp96, Hsp70, and Hsp90, a function associated with their ability to chaperone antigenic peptides and to activate antitumor cytotoxic T lymphocyte (CTL) responses (3, 4, 15). Immunization with tumor-derived HSPs initiates antitumor CTL responses via cross-presentation of their chaperoned peptides to MHC class I molecules (16, 17). By contrast, HSPs isolated from normal tissues are not effective, indicating that HSP-chaperoned peptides but not HSPs *per se* represent the tumor antigens recognized by antitumor CTLs (5). Immunization with high molecular weight stress proteins, such as Hsp110 and Grp170 (ORP150), also induced tumor-specific immune responses (10, 18, 19). Importantly, since HSP–peptide complexes act as exogenous antigens, they must be cross-presented after internalization by APCs to induce CTL responses. Therefore, focus was placed on elucidation of mechanisms including the pathway for cross-presentation as described later and identification of HSP-specific receptors.

### Role of HSPs and Their Receptors in Activation of Innate Immunity

Binder et al. first identified LDL receptor-related protein 1 (LRP1), also known as CD91, as a receptor responsible for cross-presentation of Gp96 expressed on APCs (20). Further examination revealed that CD91 was the common receptor for extracellular HSPs, including Gp96, Hsp70, Hsp90, and calreticulin (21). Some other receptors for HSP were subsequently identified. In summary, HSP receptors are divided into two groups: TLRs and SRs (1). TLR2 and TLR4 have been shown to function as receptors for Hsp60, Hsp70, and Gp96, leading to NF-κB activation. On the other hand, it has been demonstrated that Hsp70 can interact with at least four SRs: LRP1/CD91 (20), lectin-like oxidized low-density lipoprotein receptor-1 (LOX-1) (22), SR expressed by endothelial cells-1 (SREC-1) (23), faciclin, EGF-like, laminin-type EGF-like and link domain-containing SR-1 (FEEL-1) (23). Hsp70 can be bound at high affinity by these receptors and internalized, resulting in cross-presentation of the chaperoned antigen. Both Hsp90 and Hsp60 can also bind to LOX-1. Gp96, Hsp90, and calreticulin show significant binding affinity to SREC-1 and are internalized by these receptors (24).

### Role of HSPs in Activation of Innate Immunity

Matzinger proposed that the host releases endogenous signals (danger signals) that are derived from stressed or damaged cells, leading to the stimulation of immunity (the so-called danger theory) (25). Rock’s group expanded this research area (26). As danger signals, danger-associated molecule patterns (DAMPs), such as uric acid (27, 28) and pathogen-associated molecular patterns (PAMPs), such as LPS (26), have been identified.

During the course of identification of HSP receptors, in addition to their role in adaptive immunity, it has been reported that extracellular HSPs act as potent activators of innate immunity, indicating HSPs act as danger signals. Hsp60, Hsp70, Hsp90, and Gp96 have been demonstrated to stimulate TLR4 to produce inflammatory cytokines, including TNF-α and IL-12 (29). As described previously, many HSPs bind to TLR4 and stimulate production of TNF-α, IL-1β, and IL-6 via the NF-κB pathway.

### Immunogenicity of Secreted HSP

How do intracellular HSPs act as extracellular proteins? Various mechanisms have been proposed for the release of HSPs into extracellular milieu, including passive release such as that by cell necrosis caused by exposure to hypoxia, severe trauma and lytic virus infection, and active release mechanisms, Asea et al. demonstrated that IFN-γ and IL-10 induce the active release of constitutively expressed Hsc73 as well as Hsp72 from tumors (30). Moreover, Asea et al. showed that Hsp72 is also secreted in the form of exoxomes (31, 32). Mambula et al. showed that a prostatic cancer cell line secreted Hsp72 via an endolysosomal pathway (33). It would be interesting to know whether these secreted HSPs show antitumor immunogenicity.

Taking advantage of the ability of an HSP to target a chaperoned antigen peptide to APCs and elicit cross-presentation, immunotherapy using secretable forms of HSP has been developed. Yamazaki et al. demonstrated that Gp96 secreted from tumor cells carries an antigenic peptide and induces peptide-specific CTL responses (34). We also showed that tumor-derived secretable BiP elicits antigen-specific tumor immunity (35). This secreted BiP is taken up by DCs and a BiP-chaperoned antigenic peptide is cross-presented in association with MHC class I molecules, leading to CTL responses. Thus, this strategy allows tumor cells to produce their own cellular vaccine. Moreover, this strategy may be superior to a peptide vaccine strategy because single peptide-based cancer vaccines have a disadvantage. Namely, vaccination with a single peptide induces a certain HLA-restricted CTL response, thereby allowing tumor cells to escape from CTL recognition. By contrast, since a broad-spectrum antigenic peptide repertoire is associated with HSPs, induction of CTLs against multiple antigens is expected. Furthermore, a secreted HSP-based cancer vaccine is applicable for all patients, regardless of HLA restriction. Thus, gene modification of HSPs for secretion may provide a unique therapeutic approach for cancer immunotherapy.

## HSPs Navigate the Associated Antigen into Static Early Endosomes in Antigen-Presenting Cells for Cross-Presentation

As described above, immunization with purified tumor-derived HSPs or HSPs complexed with an antigen peptide/protein *in vitro* elicits tumor- or antigen-specific CD8^+^ T cell responses (4–6, 17, 35, 36). Importantly, Hsp70- and Gp96-antigenic peptide complexes facilitate antigen presentation in association with MHC class I molecules through a cross-presentation pathway (37–39). Cross-presentation is a prerequisite antigen-presentation pathway for the induction of CTL responses against viral infection and tumors. However, the precise mechanism for introduction of an exogenous antigen into a cross-presentation pathway remains unclear. Exogenous antigens can be processed through at least two distinct pathways (10, 11): one is a transporter-associated antigen-presenting (TAP)-dependent pathway, which is a classical MHC class I loading pathway, and the other is post-Golgi loading of MHC class I in endocytic compartments (endosome-recycling pathway). Cytosolic Hsp90 has been shown to translocate extracellular antigens from endosome to cytosol for TAP-dependent cross-presentation (40). In the latter pathway, internalized exogenous antigens are processed by endosomal peptidases, such as a cathepsin S, and thereafter are loaded in endocytic compartments onto MHC class I molecules that are recycled from plasma membranes (8, 41, 42).

**Figure 1:**
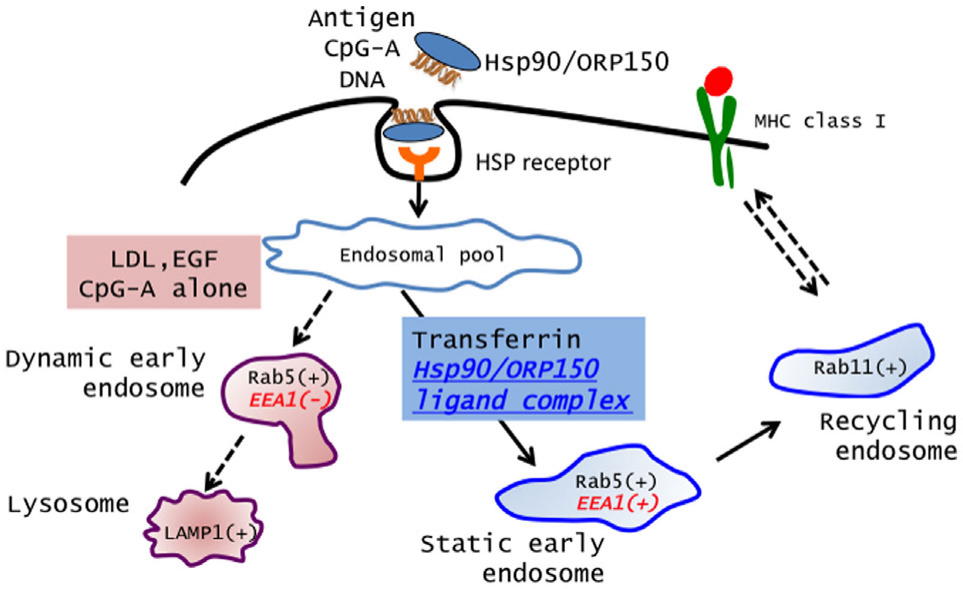
**Extracellular Hsp90/ORP150 targets chaperoned molecules into static early endosome**. Hsp90/ORP150–ligand complexes are preferentially targeted and retained in static early endosome (Rab5^+^ and EEA1^+^) but not in dynamic early endosome (Rab5^+^ and EEA1^−^).

Recently, Calderwood’s group and we showed that Hsp90 also acted as an excellent navigator for chaperoned antigens to enter the cross-presentation pathway in a murine system (9, 11, 43). Furthermore, we showed that Hsp90–peptide complex-mediated and Hsp70 family member ORP150–peptide complex-mediated cross-presentation was independent of TAP and was sensitive to membrane recycling inhibitor primaquine, indicating that sorting of peptides onto MHC class I occurs via an endosome-recycling pathway (10). We further demonstrated that the Hsp90–cancer antigen peptide complex was efficiently cross-presented by human monocyte-derived dendritic cells (Mo-DCs) and stimulated peptide-specific CTLs (12). More importantly, we showed that translocation of Hsp90–Ag complex into the “static” early endosome after endocytosis was crucial for efficient cross-presentation. Lakadamyali et al. (44) demonstrated that early endosomes are comprised of two distinct populations: one is a population of dynamic early endosome that are highly mobile on microtubules and mature rapidly toward the late endosome, and the other is a population of static early endosomes that mature much more slowly. Cargos destined for degradation, including LDL, EGF, and influenza virus, are internalized and targeted to Rab5^+^, EEA1^−^ dynamic early endosomes, followed by trafficking to Rab7^+^-late endosomes. By contrast, the recycling ligand transferrin is delivered to Rab5^+^, EEA1^+^-static early endosomes, followed by translocation to Rab11^+^-recycling endosomes. Burgdorf et al. clearly showed that a mannose receptor translocated OVA specifically into an EEA1^+^, Rab5^+^-static early endosomal compartment for subsequent cross-presentation (45). By contrast, pinocytosis conveyed OVA to lysosomes for MHC class II presentation. In addition, OVA endocytosed by a SR did not colocalize with EEA1 but colocalized with LAMP-1 in lysosomes, resulting in a presentation in the context of MHC class II molecules. We showed that the Hsp90/ORP150–peptide complex is targeted into Rab5^+^, EEA1^+^-early endosomes after internalization by human Mo-DCs, suggesting that preferential delivery to the “static” endosome is required for cross-presentation of Hsp90/ORP150–peptide complexes (10, 12). By contrast, LDL protein was targeted to the EEA1^−^, Rab5^+^, and LAMP-1^+^-dynamic early endosome–late endosome/lysosome pathway, leading to degradation. These findings suggested that Hsp90/ORP150 navigated the chaperoned antigen peptide into the static early endosome-recycling pathway, preventing extensive degradation of the peptide, followed by transfer of the peptide onto recycling MHC class I molecules within the recycling endosome. Taken together, our findings indicate that the role of Hsp90/ORP150 in cross-presentation is to shuttle the associated antigen into static early endosomes within DCs. Thus, Hsp90/ORP150 is a promising natural immunoactivator for a cancer vaccine due to its excellent ability to target human DCs and to induce specific CTLs (Figure 1).

## Hsp90-Mediated Spatiotemporal Regulation in Innate Immunity

In contrast to the idea that HSP itself acts as an endogenous danger signal, we have shown that HSP empowers the chaperoned innate ligands to activate an innate immune response via spatiotemporal regulation (46). Unmethylated single-stranded DNA containing a cytosine–phosphate–guanine (CpG) motif is recognized by TLR9, which is expressed primarily by plasmacytoid DCs (pDCs) and B cells, resulting in a large amount of IFN-α production (47, 48). Two classes of synthetic CpG-DNA have been classified: CpG-A, which stimulates to produce IFN-α by pDCs, and CpG-B, which does not. Instead, CpG-B stimulates IL-6 and TNF-α production by pDCs. It has been shown that the manner of internalization and the retention time in endosomes of these CpG-DNAs were different. CpG-A forms large multimeric aggregates with a diameter ~50 μm. By contrast, CpG-B is monomeric and does not form such high order structure. The retention of the CpG/TLR9 complex in endosomes is the primary determinant of TLR9 signaling. The multimeric CpG-A retains for longer periods of time in the early endosomes, whereas monomeric CpG-B rapidly translocates from early endosomes to late endosomes or lysosomes of pDCs (49, 50). The prolonged retention of CpG-A provides extended activation of TLR9 signaling, which leads to robust IFN-α production by pDCs.

Based on the finding that Hsp90 can target and retain chaperoned ligands within static early endosomes, we showed that human pDCs pulsed with an Hsp90–CpG-A DNA complex produce a larger amount of IFN-α than that in the case of CpG-A alone (46). Moreover, unlike human DCs, since murine conventional DCs (cDCs) express both TLR7 and TLR9 (49–51), the Hsp90-chaperoned CpG-A was retained within static early endosomes for longer periods (more than 2 h) in murine cDCs, thereby leading to sustained activation of murine cDCs and eliciting TLR9 signaling for IFN-α production. The observed IFN-α production was TLR9-dependent because cDCs isolated from TLR9 knockout mice did not produce IFN-α. By contrast, CpG-A alone was trafficked to late endosomes and lysosomes within cDCs. Interestingly, not only CpG-A but also CpG-B when chaperoned by Hsp90 could stimulate TLR9 signaling via targeting and longer retention of CpG-B within static early endosomes, resulting in the production of IFN-α. Thus, extracellular Hsp90 has the extraordinary ability to directly associated CpGs into static early endosomes, leading to IFN-α production (52). Thus, the use of extracellular HSPs may augment the effect of a cancer vaccine in combination with CpG. More importantly, extracellular HSPs might play a pivotal role in the pathogenesis of nucleic acid-mediated autoimmune diseases, such as systemic lupus erythematosus (SLE).

## Extracellular Hsp90–Self-DNA Complex Breaks Innate Tolerance via Spatiotemporal Regulation

Viral/bacterial DNA sequences contain multiple CpG nucleotides that bind and activate TLR9. By contrast, pDCs normally do not respond to self-DNA because mammalian self-DNA contains fewer such motifs, which are most likely masked by methylation (47, 48, 53). Moreover, it has been suggested that self-DNA fails to access the TLR9-containing endolysosomal compartments and is thereby unable to stimulate TLR9 (54). One of the mechanisms is that DNase easily and rapidly breaks down extracellular DNA, thereby preventing self-DNA localization into endocytic compartments. The importance of this mechanism for inhibiting autoimmune responses has been shown by the fact that mice deficient in DNase II develop SLE-like syndrome (55). Recent evidence, however, suggests that self-DNA has the potential to trigger TLR9 when self-DNA is engaged TLR9 appropriately (56, 57). Mammalian DNA in the form of chromatin-containing immune complexes could stimulate TLR9 in association with B-cell receptors in an *in vitro* study (58, 59). Breakdown of innate tolerance to self-nucleic acids occurs when tissue injury or necrosis causes the release of some endogenous molecules, including antimicrobial peptides, such as LL37 (60) and nuclear protein HMGB-1, which help to promote stabilization and delivery of associated innate ligands, including nucleic acids, into early endosomes (61). These molecules have been shown to play a critical role in the initiation of autoimmune diseases through the production of IFN-α.

**Figure 2:**
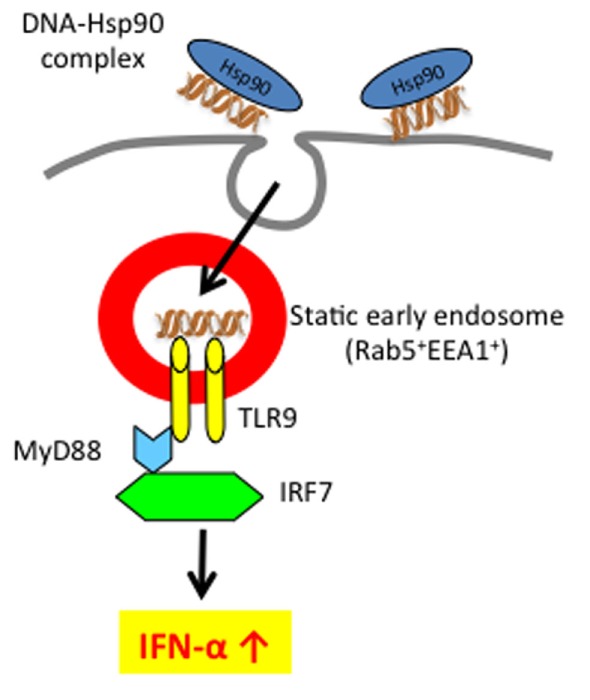
**Extracellular self-DNA stimulates pDC to produce IFN-α when chaperoned by Hsp90 via spatiotemporal regulation**. Hsp90–self-DNA complex found in SLE patient’s serum is targeted to pDCs and retained for longer periods within early static endosome via action of Hsp90, leading to production of IFN-α.

As described above, since Hsp90 can bind DNA (62), we examined whether Hsp90 targets self-DNA into static early endosomes, resulting in IFN-α production by human pDCs (46). Upon Hsp90-mediated enforced endosomal translocation as well as longer retention, human self-DNA could activate DCs via TLR9 to produce IFN-α. Thus, Hsp90 regulates TLR9-mediated responses through spatiotemporal regulation of Hsp90-chaperoned ligand complexes (Figure 2). Therefore, targeting TLR9 and modulating TLR9 signaling using Hs90 inhibitors or siRNA to Hsp90 have emerged as important strategies for the treatment of self-nucleic acid-related autoimmune diseases, including SLE. In the following section, we will discuss the emerging topic for the role of extracellular and intracellular Hsp90 in the pathogenesis of SLE.

## Role of Hsp90 in SLE

### Extracellular Hsp90 Plays a Pivotal Role in the Pathological Condition of SLE

Systemic lupus erythematosus is a prototype of autoimmune disease characterized by the production of autoantibodies specific for self-nuclear antigens, such as dsDNA and RNA-containing antigens, including Sm and RNP (63, 64). Recently, it has been demonstrated that a prominent feature of SLE is increased expression of type I IFN-regulated genes in blood cells, which is often associated with increase of IFN-α in the serum (65–67). Moreover, it has been shown that elevated IFN-α in SLE patient’s serum accelerates the disease severity of SLE and is associated with disease activity (68–71).

Plasmacytoid DCs are major producers of IFN-α (72), and are decreased in number in the blood but are abundant in skin and lymph nodes (67). In SLE patients, immune complexes consisting of autoantibodies bound to self-DNA and RNA can stimulate production of IFN-α through TLR9 and TLR7 after uptake via Fc receptors (73). The pathogenic role of IFN-α in SLE is mediated partly by its ability to induce immune activation, including a positive feedback loop that induces plasma cell maturation and increases autoantibody production (74). The role of IFN-α in this disease has now been confirmed in lupus-prone mouse models (75, 76).

Previous studies have demonstrated the presence of autoantibodies to Hsp90 (77, 78) and showed enhanced expression of Hsp90 in peripheral blood mononuclear cells of patients with active SLE (79, 80), suggesting a role of Hsp90 in SLE. In addition, Hsp90 has been shown to localize both in the cytoplasm and nucleus (81). Under stressful conditions, cytosolic Hsp90 translocates to the nucleus (82). This suggests that Hsp90 may bind self-DNA within the nucleus. When cells undergo necrosis, self-DNA associated with endogenous Hsp90 can be released into the extracellular milieu and might trigger IFN-α production by pDCs. In fact, we found that serum levels of Hsp90 were significantly increased in patients with active SLE compared with the levels in patients with inactive SLE or other autoimmune diseases (83). Serum Hsp90α levels increased with increase in SLEDAI score. Moreover, serum Hsp90α in patients with SLE was significantly decreased after treatment. Of interest, extracellular self-Hsp90 found in SLE sera could stimulate IFN-α production by pDCs. We also found that extracellular self-Hsp90 associated with self-DNA or an anti-DNA antibody–self-DNA complex (Figure 2). This Hsp90–self-DNA complex or Hsp90–anti-DNA autoantibody–self-DNA complex might be efficiently endocytosed and targeted to early endosomes via the action of Hsp90, leading to the robust IFN-α production observed in SLE sera (46). Moreover, immunodepletion of extracellular Hsp90 from SLE serum decreased IFN-α production by pDCs (83), indicating that depletion of Hsp90 might induce remission and prevent end-organ damage. Thus, control of deregulated pDC activation and IFN-α production provides an alternative treatment strategy for SLE.

Collectively, extracellular Hsp90 plays a crucial role in the disease activity of SLE and that Hsp90 inhibitors have promise for the treatment of IFN-α-mediated autoimmune diseases including SLE.

### Hsp90 Chaperones TLR7/9 to Sense Self-Nucleic Acids

The localization of TLR7/9 is unique, because they reside in the ER at the quiescent stage and traffic to endosomes only upon ligand recognition (60). The underlying mechanism for adequate transportation of these TLRs has been investigated. Recently, Unc93B1, a multitransmembrane ER-resident protein, has been shown to associate with and deliver TLR7/9 from the ER to endosomes, where TLR7/9 recognize their ligands (84). In addition, gp96, an ER-resident HSP, has been shown to be a master immune chaperone for both cell surface and intracellular TLRs, including TLR1, 2, 4, 5, 7, and 9 (85). Furthermore, ER luminal protein PRAT4A (also known as CNPY3) also translocates TLR1, 2, 4, 5, 7, and 9 to either cell surfaces or endolysosomes (86). Recently, it has been shown that gp96 cooperates with PRAT4A for the trafficking of theses TLRs (87).

**Figure 3:**
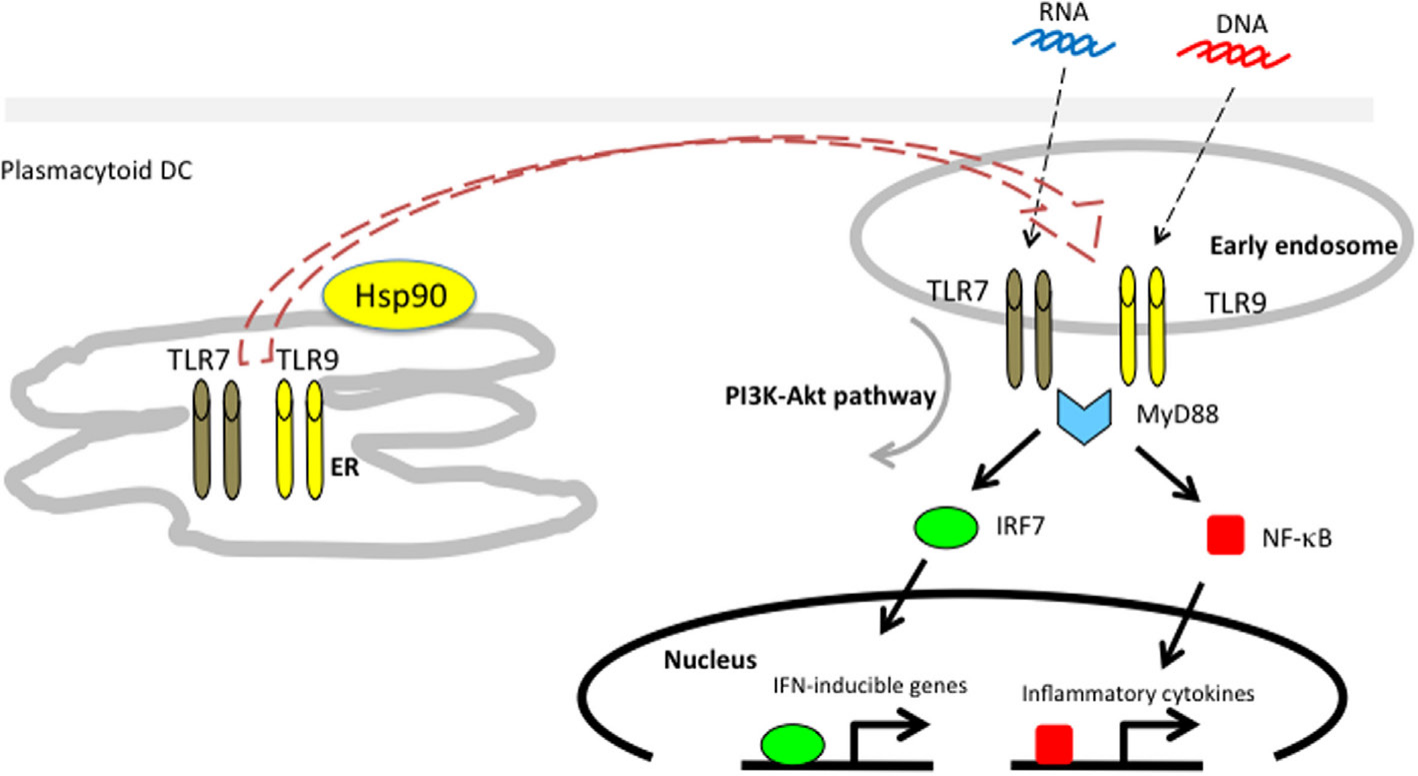
**Hsp90 associates with toll-like receptors 7/9 and mediates self-nucleic acid recognition in plasmacytoid DCs**. Hsp90 has a spatial interaction with TLR7/9 and chaperones them from the ER to the early static endosome.

We showed that endogenous Hsp90 also had a crucial role on the production of IFN-α in response to CpG-A by human pDCs using the Hsp90 inhibitor 17-AAG (83). The 17-AAG treatment dramatically inhibited the production of IFN-α. Further studies revealed that Hsp90 interacted with TLR7/9 and, more importantly, that Hsp90 chaperoned TLR7/9 from the ER to the early endosome (Figure 3). Inhibition of Hsp90 by 17-AAG resulted in disruption of the interaction of Hsp90 with TLR7/9, leading to inhibition of IRF7 phosphorylation and nuclear localization, which impaired the production of IFN-α. Thus, Hsp90 is a cytosolic chaperone for TLR7 and TLR9 and is essential for TLR7/9-mediated innate immune responses (83).

### Possible Therapeutic Use of an Hs90 Inhibitor in SLE

The involvement of IFN-α in the pathogenesis of SLE indicates the possibility of therapeutically targeting either IFN-α or the mechanisms leading to IFN-α production. We examined the effect of Hsp90 inhibitor in SLE-prone MRL/lpr mice (83). Treatment with the Hsp90 inhibitor 17-DMAG significantly ameliorated disease activity as well as histopathological findings in diseased mice. The dramatic decrease in severity of SLE seemed to be due to simultaneous blocking of TLR7 and TLR9 signaling by the Hsp90 inhibitor. Shimp et al. also showed that administration of 17-DMAG ameliorated SLE symptoms (88). Interestingly, similar to patients with SLE, serum Hsp90 in SLE-developed mice was clearly decreased in the 17-DMAG-treated group compared with that in the untreated group. Thus, our results indicate that both intracellular Hsp90 and extracellular Hsp90 play a crucial role in the pathogenesis of SLE and that Hsp90 inhibitors have promise for the treatment of IFN-α-mediated autoimmune diseases including SLE.

## Future Prospects of HSPs in Immunology

Our understanding of the mechanisms by which extracellular HSPs play pivotal roles in the regulation of innate immunity and adaptive immunity has been progressing at a very fast pace. We here focus on the extraordinary ability of Hsp90/ORP150 to target chaperoned molecules into static early endosomes after being taken up by DCs. Several receptors specific for HSPs expressed on DCs have been identified (21, 43). However, it has been unclear whether these receptors contribute to the sorting of HSP-chaperoned molecules into static endosomes. Moreover, it should be clarified whether HSPs other than Hsp90/ORP150 act in a fashion similar to Hsp90/ORP150. By clarifying these issues, HSPs will open innovative therapeutic opportunities in cancer and autoimmune diseases.

## Author Contributions

YT wrote the paper. AY, NT, and KS conducted experiments and analyzed data.

## Conflict of Interest Statement

The authors declare that the research was conducted in the absence of any commercial or financial relationships that could be construed as a potential conflict of interest.
